# The influence of anxiety on quality of life among patients with an indication for cataract surgery: A influência da ansiedade na qualidade de vida dos pacientes com indicação de cirurgia de catarata

**DOI:** 10.1590/1516-3180.2014.9510109

**Published:** 2014-11-28

**Authors:** Larissa Fazzi, Fernanda Martinho Dobrianskyj, José Ricardo Abreu Reggi, Victor Henrique Oyamada Otani, Thais Zelia Santos Otani, Ricardo Riyoiti Uchida

**Affiliations:** I Undergraduate Medical Student, Faculdade de Ciências Médicas da Santa Casa de São Paulo (FCMSCSP), São Paulo, Brazil.; II MD, PhD. Attending Physician, Department of Ophthalmology, Faculdade de Ciências Médicas da Santa Casa de São Paulo (FCMSCSP), São Paulo, Brazil.; III MD, MSc. Attending Physician, Department of Psychiatry, Faculdade de Ciências Médicas da Santa Casa de São Paulo (FCMSCSP), São Paulo, Brazil.; IV MD. Attending Physician, Department of Psychiatry, Faculdade de Ciências Médicas da Santa Casa de São Paulo (FCMSCSP), São Paulo, Brazil.; V MD, PhD. Associate Professor, Department of Psychiatry, Faculdade de Ciências Médicas da Santa Casa de São Paulo (FCMSCSP), São Paulo, Brazil.

Anxiety is defined as an unpleasant, diffuse and vague feeling^1^ that may affect patients’ quality of life. There is growing concern regarding anxiety within medicine, especially given that humanization is becoming a more important issue following the large technological developments that have been seen within the health sciences. The main objective has gone beyond merely controlling the symptoms, decreasing mortality and increasing life expectancy, since the psychological aspects of health and illness are ever more greatly valued.

The present study sought to assess the influence of anxiety on the quality of life of patients with cataracts acquired in the first eye after reaching the age of 50 years and who did not present any severe cognitive deficit. We evaluated 52 patients with cataracts (21 males), with an average age of 72.11 years (± 9.23), on the day that the cataract surgery was indicated at the ophthalmology outpatient clinic of Santa Casa de Misericórdia de São Paulo. The Inventory of Anxiety Trait-State (IDATE) scales were used to assess anxiety and the World Health Organization Quality of Life (WHOQOL)-Bref questionnaire was used to assess quality of life.

The IDATE-t average score was 38.88 (± 11.15). The separate quality-of-life domain scores were: a) general = 3.42 (± 0.67); b) physical = 3.06 (± 0.70); c) environment = 3.43 (± 0.59); d) psychological = 3.74 (± 0.58); e) social = 3.84 (± 0.46). The IDATE-t total scores correlated negatively with the following quality-of-life domains: general [r = -0.33, P = 0.01]; physical [r = -0.43, P < 0.01]; environment [r = -0.44, P < 0.01] and psychological [r = -0.67, P < 0.01]. On the other hand, there was no correlation between the IDATE scale and the “social domain”, which encompasses personal relationships, social support and sexual activity.

The present study demonstrates that there is a high psychosocial burden among patients with cataracts who are assessed through the IDATE-t and WHOQOL scales. Additionally, it shows that these patients’ quality of life has an inverse relationship with anxiety levels. This finding corroborates previous studies that have demonstrated low quality-of-life scores among patients with cataracts^2^ and the negative impact of anxiety disorders on the quality of life of patients undergoing cataract surgery.^3^
